# Bio-properties of Saba banana (*Musa ‘saba’*, ABB Group): Influence of maturity and changes during simulated *in vitro* gastrointestinal digestion

**DOI:** 10.1038/s41598-020-63501-x

**Published:** 2020-04-21

**Authors:** Florencio Collado Reginio, Wei Qin, Sunantha Ketnawa, Yukiharu Ogawa

**Affiliations:** 1 Graduate School of Horticulture, Chiba University, 648, Matsudo, Matsudo City, Chiba 271-8510 Japan; 2 Institute of Food Science and Technology, College of Agriculture and Food Science, University of the Philippines Los Baños, College, Laguna, 4031 Philippines

**Keywords:** Plant sciences, Nutrition

## Abstract

Saba banana, a popular fruit crop grown in Southeast Asia, is an economical source of a variety of beneficial agents. This study examined the variations in total phenolic, flavonoid, and antioxidant activities of five maturity stages of Saba banana, and their changes during simulated *in vitro* gastrointestinal digestion as affected by varying structural compositions. Antioxidant activities were evaluated using ferric reducing antioxidant power (FRAP), metal ion chelating (MIC) activity, and 2,2-diphenyl-1-picrylhydrazyl (DPPH) and 2,2′-azino-bis(3-ethylbenzothiazoline-6-sulfonic acid) (ABTS) assays. Results of DPPH and ABTS were compared in terms of TEAC (Trolox Equivalent Antioxidant Capacity) and VCEAC (Vitamin C Equivalent Antioxidant Capacity) values. Bio-properties were found to be highest in mature green stage with values slightly decreased as ripening proceeded. Simulated digestion showed a continuous increase in total phenolic with comparatively faster release in structure-less state (slurry) than samples with intact structure (cut). The trend of antioxidant activities was increased in the gastric phase and then decreased at the onset of intestinal phase, except for MIC which showed a reverse effect. Our study indicated that the bio-properties of Saba banana were affected by maturity and modifications in its physical structure and composition could influence the release behaviors of food components during simulated digestion.

## Introduction

Owing to its increasing demand and whole-year availability, banana is considered as one of the world’s most important fruit crops in terms of commercial production and one of the most consumed staple food commodities after major cereals^1,2^. An estimated of 19.2 million tons of banana had been exported globally in 2018 which accounted to the strong supply growth from the two leading exporters, Ecuador and the Philippines^3^. Among the economically important banana cultivars in the Philippines, foremost of which is Saba (*Musa ‘saba’*[*Musa acuminata* × *Musa balbisiana*]), an ABB genome group, which accounts to more than 25% of the country’s banana production. This banana variety can be utilized at all stages of maturity either raw or cooked and is used primarily for manufacturing various food products such as condiments (banana ketchup), snacks (banana chips), viands, and desserts^4^. Saba banana is also relished in other Southeast Asian countries^5^ but is gaining popularity in Latin America^2^ and Southern Nigeria^6^.

Banana is not only an important source of starchy staple food but could also exert a beneficial effect on human health. It contains phytonutrients, including vitamins and different classes of phenolic compounds^2,7^. In plants, phenolic compounds may exist in free, soluble conjugated (esterified), and insoluble-bound forms^8,9^. Research studies have shown that bound phenolics have demonstrated a significantly higher antioxidant capacity compared to free phenolics in numerous *in vitro* antioxidant assays carried out^10^. Insoluble bound phenolics are localized in the cell wall matrix of plant cells which are covalently bonded to cell wall components such as cellulose, pectin, and structural proteins^9^ and can be released after acidic or alkaline hydrolysis^8^. The beneficial effect of these phenolic compounds as source of natural antioxidants is associated with health protective properties. The protection mechanism is generally by inhibiting the formation of free radical species and repairing oxidative damage, thus preventing the development of various chronic and degenerative diseases^11,12^.

The antioxidant capacity of fruit is determined by a mixture of various antioxidant compounds with different action mechanisms; therefore, it is necessary to combine more than one method in order to provide a broader picture of the antioxidant capacity of foodstuffs^13^. The most widely used methods being 2,2′-azino-bis(3-ethylbenzothiazoline-6-sulfonic acid) (ABTS) and 2,2-diphenyl-1-picrylhydrazyl (DPPH) assays, among others such as oxygen radical absorbance capacity (ORAC), ferric reducing antioxidant power (FRAP) assay^14^, and metal ion chelating (MIC) activity. A well-established parameter to express the antioxidant activity of biological sample as equivalents of standard antioxidant in this respect being Trolox Equivalent Antioxidant Capacity (TEAC)^15^ and Vitamin C Equivalent Antioxidant Capacity (VCEAC)^14^.

The bioaccessibility of antioxidant compounds during gastrointestinal digestion is crucial for their absorption and bioavailability. Thus, *in vitro* studies, even though typically constituting of only a static model of digestion, have been developed to allow the holistic understanding of the actual effects of nutritional ingredients on the living body and the changes and release of antioxidant compounds from the food matrix^16^ as affected by the composition and structural features of food under simulated gastrointestinal conditions. It is a faster and more cost-effective method to simulate the natural digestive process and rapidly screen food products for their estimated biological activity^17^. Though it cannot perfectly actualize the highly complex physiological events during digestion, it has been demonstrated that the evaluation of bioaccessibility through *in vitro* models can be well correlated with results from *in vivo* studies and animal models^18^, which was patterned after the gastrointestinal digestion conditions of a healthy adult human.

Nutrient bioaccessibility during gastrointestinal digestion process varies for the same food depending on processing conditions and presence of other components^19^. Mechanical processes such as grinding or cutting could either disrupt or retain the cellular structure of food^20^ which may have an impact on the release and absorption of nutrients. The presence of intact cells in the food matrix have been reported to survive digestion in the upper gastrointestinal tract and that mastication could bring damage to the cells of plants which made nutrients bioaccessible^19^. On the other hand, the rise and loss of fruit components or attributes (cell integrity, acids, sugars, pectin) during the course of ripening^21,22^ may also bring significant effect on the transition of food compounds during digestion. However, not many studies have determined how varying physical structures and maturation changes in fruits affect the release of bioactive compounds during digestion.

The bio-properties of Saba banana have been the subject of limited studies that focused mainly on the ripe stage of maturity^23^ and the content of few extractable free phenolics, ignoring the bound fractions^11^. Subsequently, a comprehensive review by Singh *et al*.^2^ summarized the previous researches on bioactive compounds of different banana cultivars including ABB genome group of Saba banana. However, insufficient data exist on the bio-properties of mature unripe counterpart and the content of bound phenolics. The analysis of bound phenolics will provide a better estimate of the food’s actual contribution on biological activities, and the determination of antioxidant capacity of mature green stage will present a basis in the selection of optimum maturity stage that may have great potential as source of bioactive compounds for eventual production of functional products. Moreover, since Saba banana has become one of the important fruit crops for consumption, observation of the digestive fate of its compounds is important to assess their chemical and physical stability in the varying conditions of the gastrointestinal tract.

Given the above, this study aimed to determine the variations in free and bound phenolics, flavonoid, and antioxidant activities of different maturity stages of Saba banana and investigate the changes undergone by phenolics and antioxidant activities during simulated *in vitro* gastrointestinal digestion. The effect of varying physical structures of food was also evaluated through preparation of homogenized slurry and unhomogenized cut samples representing structure-less and intact cellular structure, respectively. Additionally, the comparability of DPPH and ABTS assays, expressed as TEAC and VCEAC values, was also evaluated. The study will provide information on the quality attributes of Saba banana in terms of its biochemical properties and its potential health benefits upon subjecting to the physical, enzymatic, and chemical processes of simulated digestion.

## Results

### Bio-properties of fresh Saba banana during maturation

**Figure 1:**
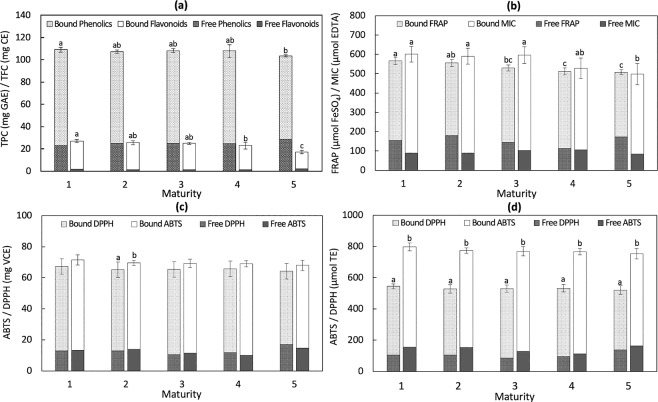
Bio-properties of fresh Saba banana at different maturity stages (*n* = 5). For a and b, mean values with different lowercase letters for the same parameter indicate significant differences between maturity stages (*p* < 0.05). For c and d, mean values with different lowercase letters indicate significant differences between antioxidant assays (*p* < 0.05).

A decrease in total phenolic content (TPC) and total flavonoid content (TFC) was observed as maturity progressed, with significant differences between the first and last stages (Fig. 1a). From 109.06 ± 2.11 mg in the mature green stage, TPC decreased to 103.47 ± 1.02 mg in stage 5; however, no significant difference was observed between stage 1 and middle stages (2, 3, and 4). TFC also showed no significant difference among the initial stages (1, 2, and 3) and accounted on average for 18–26% of TPC ranging from 19.42 ± 1.55 mg to 28.70 ± 1.41 mg. Bound phenolics were on the average around 3-fold higher than free fractions. Similarly, around 90% of TFC was obtained from bound fractions.

Antioxidant activities followed a similar trend to that of TPC as ripening proceeded (Fig. 1b–d). The antioxidant activity of free phenolic was also found to be lower compared to that of bound phenolic. The Saba banana showed FRAP and MIC values ranging from 498.33 ± 54.35 µmol to 601.04 ± 40.19 µmol. Comparing the two radical scavenging assays, antioxidant capacities by ABTS (753.60 ± 34.24 µmol to 797.5 ± 24.9 µmol TEAC and 68.02 ± 3.39 mg to 71.53 ± 3.24 mg VCEAC) was consistently higher than antioxidant capacities by DPPH (519.98 ± 27.19 µmol to 545.12 ± 14.80 µmol TEAC and 64.11 ± 3.40 mg to 67.25 ± 1.86 mg VCEAC). Significant differences between TEAC of ABTS and DPPH assays were observed while, in general, no significant difference was determined in VCEAC.

### Bio-properties of different structural states of Saba banana during simulated *in vitro* digestion

**Figure 2:**
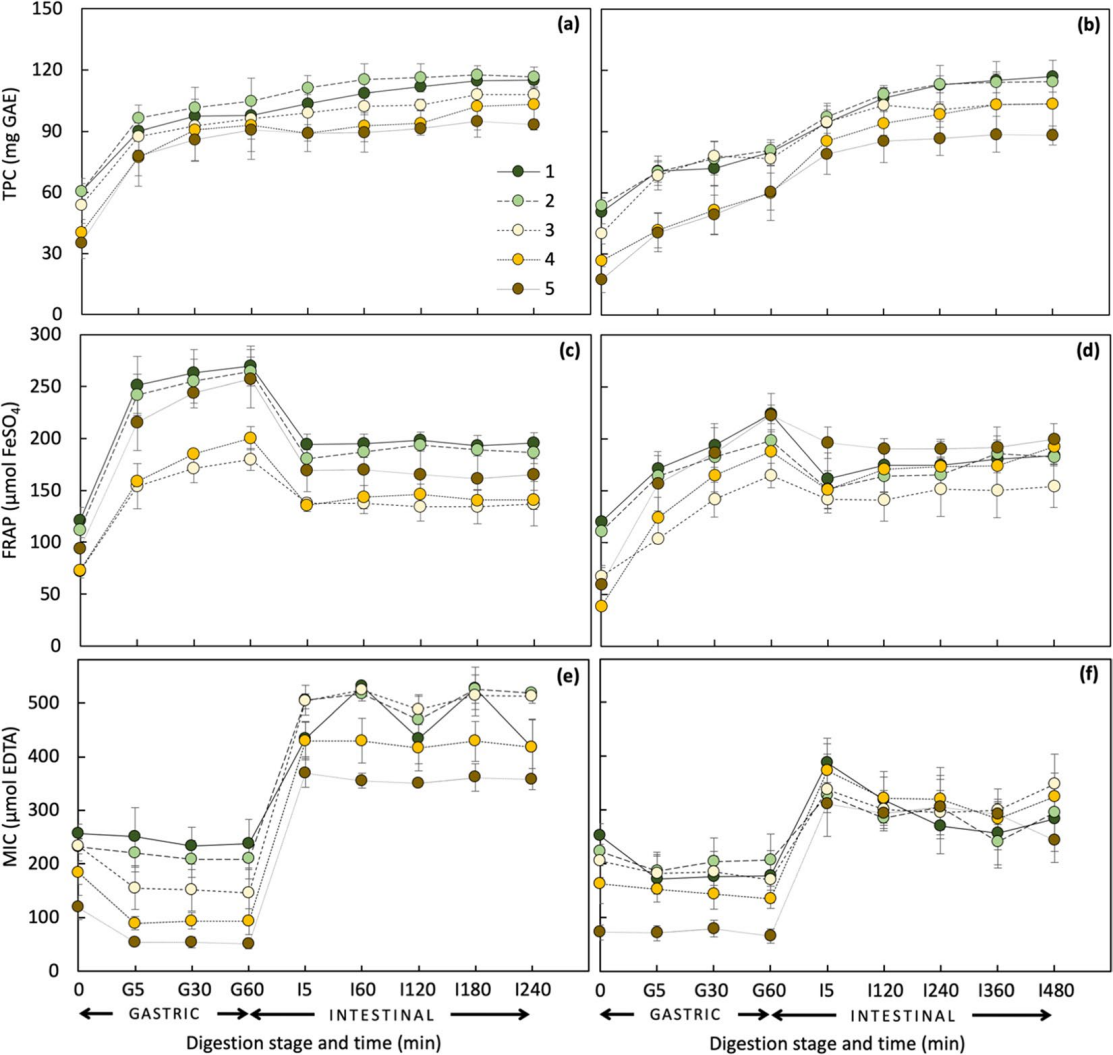
TPC, FRAP, and MIC values of digested fractions of slurry (**a,c,e**) and cut (**b,d,f**) Saba banana at different maturity stages (*n* = 3).

**Figure 3:**
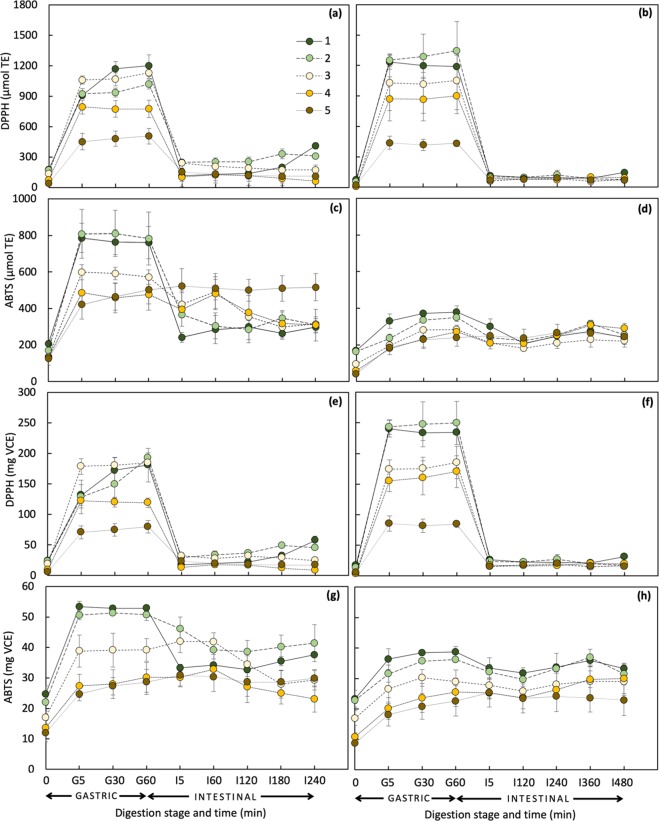
TEAC and VCEAC values of digested fractions of slurry (**a,c,e,g**) and cut (**b,d,f,h**) Saba banana (*n* = 3).

At time 0, stages 1 and 2 of both slurry and cut states of digested Saba banana samples showed the same behavior as that of fresh which found to have higher values of TPC and antioxidant activities than other maturity stages (Figs. 2,3). From initial (time 0) to gastric phase (G60) of simulated digestion, there was on the average a 1.5- to 3-fold increase in the amount of total phenolics in both states with the highest increment observed in ripe stages (Fig. 2a,b). Following gastric digestion, TPC, in general, had a continuous increase over the duration of digestion and was released more rapidly in slurry than cut state.

A comparable trend of increasing antioxidant activity values with increasing digestion time during gastric phase was observed, except for MIC. From time 0 to G60, FRAP values of slurry samples increased more than 2-fold while values for cut samples varied from 1.8- to 4.9-fold (Fig. 2c,d). Both slurry and cut states were found to have higher percent increment in ripe stages (4 and 5) than in mature green counterpart. The TEAC values of slurry samples on the basis of the ability to scavenge DPPH^•^ were around 2- to 4-fold higher than that of ABTS^•+^ in the gastric phase while cut samples showed a much wider gap (3- to 46-fold) (Fig. 3). This trend was consistent even in VCEAC exhibiting a 3- to 6-fold and a 6- to 28-fold greater antioxidant capacity of DPPH than ABTS for slurry and cut samples, respectively. Interestingly, MIC values from time 0 to G60 of slurry samples dropped as maturity advanced (7% decrement in stage 1 to 57% in stage 5) (Fig. 2e). The cut samples, on the other hand, showed no definite trend (Fig. 2f).

In contrast with TPC, antioxidant activities were shown to decrease at the onset of intestinal digestion (I5), except for MIC. From G60 to I5, a percent decrement ranging from 12–30% was observed in FRAP and more than 60% in DPPH assay. Surprisingly, different from other maturity stages, ABTS assay values of stage 5 showed a percent increment of 2–12% from gastric to intestinal phase. Similarly, MIC exhibited a reverse trend of results compared to those observed in other antioxidant activity measurements. A sharp increase ranging from 1.6- to 7-fold was detected with the last stage of maturity having the highest increment.

At the end of simulated digestion (I240 and I480 for slurry and cut samples, respectively), similar to the results of fresh Saba banana, antioxidant capacities detected by ABTS assay of slurry samples from time 0 showed on the average a higher percent TEAC increment (44–305%) than DPPH assay (24–177%) while no significant difference was observed in VCEAC. Both assays had the highest increment in the last stage of maturity. The cut samples, on the other hand, showed a different trend having higher percent increment values of both TEAC and VCEAC in DPPH assay.

### Correlation analysis

**Figure 4:**
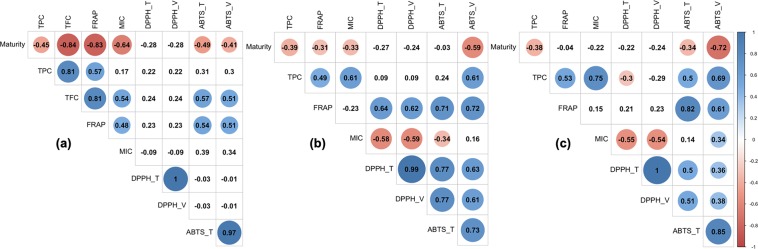
Correlation analysis between different parameters (*p* < 0.05) of fresh (**a**) and digested (slurry [**b**] and cut [**c**]) Saba banana. T = TEAC; V = VCEAC.

The maturity of fresh Saba banana showed significant negative linear correlation (*p* < 0.05) with TPC, TFC, and antioxidant activities, except for DPPH (Fig. 4a). The correlation between the release of antioxidant compounds and maturity during *in vitro* digestion in homogenized slurry sample showed almost the same result as fresh with no significant relationship for both TEAC and VCEAC of DPPH assay (Fig. 4b). However, in unhomogenized cut state, only TPC and TEAC and VCEAC of ABTS was found to have significant correlation with maturity (Fig. 4c). In both fresh and digested fractions, only FRAP was found to have a moderate to strong correlation with TPC among the antioxidant activity assays. There were more linear correlations of TPC with antioxidant activity in digested samples than in fresh state; the highest of which was obtained in cut samples. In addition, antioxidant activities showed positive correlation to each other, except for MIC. TEAC and VCEAC measured by ABTS assay were significantly correlated to antioxidant activities by DPPH assay in digested fractions while no correlation was observed in fresh sample.

## Discussion

The determination of total phenolic content as free and bound forms in Saba banana revealed that, between the two, a major fraction was contributed from bound phenolics. A previous evaluation of banana pulp showed that it contained significant levels of cell wall bound phenolics such as anthocyanidins^7^, quercetin, and cyanidin-3-*O*-glucoside chloride^24^. Since bound phenolics are bound to insoluble macromolecules, they have different pathways and absorption mechanisms in the gastrointestinal tract when compared to that of free phenolics. Free phenolics has partial release in the mouth and absorb in the small intestine; however, bound phenolics move directly to the colon where they undergo fermentation by the gut microbiota, thereby releasing the bound phenolics^9^. Nevertheless, both free and bound fractions are sources of natural antioxidant compounds, as shown by their reactions and activities in different antioxidant assays performed.

The observed high phenolic contents and antioxidant activities of mature green stage which then declined as ripening proceeded was comparable to previous studies^7,25,26^. This could be accounted to the different biochemical, physiological, and structural reactions and modifications happen as the fruit undergoes important changes during maturation process, thus affecting the contents of polyphenols and their antioxidant activities. Such modification could be associated to the oxidation of polyphenols by polyphenol oxidase (PPO)^27^ which results to polyphenols cross-linked with other polyphenols, carbohydrates, or proteins^28^. The activity of PPO during banana ripening has been widely studied and shows varying trends of results in previously conducted researches. Montgomery and Sgarbieri^29^ found a 35% decrease PPO activity in ripening banana pulp. Giami and Alu^30^, reported around 4-fold increase in PPO with ripening while Young^31^ observed no change in the activities of several banana enzymes as ripening proceeded. Though differences in the previous results are evident, this cannot deny the fact that PPO is still active even during the late stages of ripening which could affect the contents of phenolic compounds in Saba banana during storage.

Fruit maturation involves high metabolic activity which usually requires physiological mechanisms of defense^21^. This is important as ripening in most fruits like banana encompasses the conversion of insoluble protopectin into water-soluble pectin, which softens the texture of the fruit and eventually results in cell wall deterioration^22^. An important mechanism by which the plants could strengthen their cell walls, providing both physical and chemical barriers, and defend themselves against invasion of pathogens during this period is through the integration of phenolic esters into the cell walls. Additionally, these phenolic esters could also prevent the action of reactive oxygen species on cell membrane damage. These caused the observed decrease in the phenolic acid content of date palm during ripening^32^, which could also account to the trend of results of this study.

Our study measured antioxidant activities by the ability to scavenge free radicals (DPPH^•^ and ABTS^•+^), chelate ferrous ions (MIC) and reduce ferric iron (FRAP). The results obtained comparing the two free radical assays showed the same trend of change during simulated digestion; however, values of DPPH assay possibly underestimated the antioxidant capacity and ABTS assay showed better values, which was in agreement with previous findings^13,14^. The solubility of ABTS^•+^ in aqueous solution allows it to measure hydrophilic compounds^15^, which made up >90% of the total antioxidant capacity in most fruits^33^. Aside from the difference in reaction media, the variations in the composition of radicals used and their reaction mechanisms could contribute to the observed discrepancies in the readings of the two scavenging assays. ABTS assay is an electron transfer reaction while DPPH is based on the normal hydrogen atom transfer between antioxidants and nitrogen radical. The creation of a more stable and less transient nitrogen radicals, instead of peroxyl radical which is highly reactive, caused many antioxidants to react slowly or may even become inert to DPPH^•^^34^, unlike ABTS^•+^ in which different antioxidant compounds donate one or two electrons to reduce the radical cation^35^. This could be the reason for the stronger correlation of ABTS assay with maturity and TPC of the digested samples, which was in agreement with previous study^14^. However, the obtained antioxidant capacity values in the present study were found to be higher compared to the previously published data about banana owing to the difference in cultivar used and the extraction method applied. Proteggente *et al*.^36^ reported a TEAC value of 181 µmol/ 100 g fresh weight (FW) of banana varieties from Caribbean region using ABTS assay. Zang *et al*.^37^ classified banana as having low antioxidant activity with VCEAC reading of <30 mg/100 g FW. Both studies did not determine bound phenolics; the reason for the lower TEAC and VCEAC values. This was also true for the result of FRAP activity which was higher by more than 3-fold than the reading of the previous study^36^. On the other hand, banana was found to have the highest ferrous ion chelating power (>50% in 100 mg fruit/mL) among the tropical fruits studied by Lim *et al*.^38^.

Regarding the low correlation between antioxidant activities and TPC of fresh Saba banana extract, this could indicate that phenolic compounds were not the sole contributors to the antioxidant capacities of the fruit crop. It is worth noting that other secondary metabolites with antioxidant potential might be accountable in enhancing the antioxidant activities of Saba banana. This includes vitamin C, β-carotene, and vitamin E^11^. On the average, banana pulp at ripening stage contains ascorbic acid in the range of 6.9–10 mg/100 g FW, a wide variation of β-carotene levels ranging from 92 to 636 µg/100 g dry weight^2^, and vitamin E content in the range of 0.06–0.52 mg/100 g FW^39^.

Based on the results of simulated digestion, gastric and intestinal conditions affected the release of TPC and antioxidant activities of Saba banana. Though the two digestive phases showed different trend of results, it is evident that the action of digestive enzymes contributed to the release of bioactive compounds, which could make them bioaccessible, and therefore potentially bioavailable. In general, TPC and antioxidant activities were increasing during the gastric phase, except for MIC. The acidic pH environment in the gastric stage may induce the release, hydrolysis and/or transformation of phenolic compounds^40^ and these compounds are stable under acid conditions^41^. This was supported by previous *in vitro* studies which reported high stability and released rate of flavonoids and phenolic acids under stomach conditions^42,43^ resulting to an increase in total phenolics. However, during the intestinal digestion, a decreasing trend in the values of antioxidant activities was observed, as also shown in previous *in vitro* digestibility studies such as apples^18^, lettuce^44^, and some edible flowers^35^. While small intestine is considered as the major site for free phenolics absorption, the change in pH condition during intestinal digestion affected the amounts of released phenolic compounds. There was a high possibility that a proportion of the phenolic compounds, which are sensitive to mild alkaline conditions of the small intestine, were transformed into different structural forms with different chemical properties, and thus affecting their biological activity^42^. An example is the biochemical transformation of flavylium cation of anthocyanidin to less stable chalcone and carbinol pseudobase forms even at near-neutral pH^45^, which eventually leads to degradation of anthocyanin^42,46^. Similarly, flavonols, such as quercetin, have been reported to undergo oxidation, hydroxylation, and ring cleavage at medium pH values in a time-dependent manner resulting to the formation of complex product profiles^47^. The low chemical stability of these compounds, as a function of their chemical structure, in the mild alkaline pH condition of the small intestine could cause their degradation and inactivity^46^. In addition, pH could affect the racemization of molecules, possibly changing the composition of the compounds and altering their biological reactivity, as pH at intestinal stage could increase racemization in some compounds, thus affecting their antioxidant activity^35^. In contrast with the results of reducing ability of antioxidant compounds, iron chelating properties showed high stability in near-neutral pH concentrations. Most flavonoids were observed to have high chelating activity in pH 6.8 and 7.5 while low to no activity at acidic pH (4.5). This could be accounted to the different proton dissociation reactions of hydroxyl groups of flavonoids in various pH which found to have high efficiency at neutral than acidic conditions^48^.

The sudden decrease of antioxidant activity during intestinal digestion still requires detailed investigation, as discussion about this phenomenon is deficient, which according to some studies may also be brought by digestive enzyme interaction with polyphenols^46^. Nevertheless, TPC values remained statistically the same after simulated intestinal digestion as compounds such as gallic acid can tolerate neutral to harsh alkaline conditions in the small intestine^45^. Other possibilities that could explain the observed trends are: (a) the phenolics detected during simulated intestinal digestion were only metabolites without antioxidant property^43^ and (b) the antioxidant activity values were affected by the sensitivity of the assays to pH changes which could also explain the surprisingly higher values of DPPH than ABTS during the gastric phase. Unlike ABTS assay, DPPH assay is more sensitive to pH^49^ and is found to have high percent inhibition at pH less than 4; since at pH higher than 4.5, possibility of deprotonation of gallic acid could occur resulting to decreased inhibition of DPPH^•^ by having no ability to donate hydrogen atom^50^. DPPH assay is also sensitive to oxygen which could explain the lower TEAC and VCEAC values of slurry than cut state. The diradical property of oxygen incorporated during homogenization could react with DPPH^•^ directly with the presence of light energy, and thus decrease the absorbance of DPPH^51^.

With regard to the differences in the observed trend between cut and slurry samples, their varying physical structures could affect the release of bioactive compounds. The structure of food is known to have an important role on the bioaccessibility of nutrients^52^. Manipulating the structure of food to control the release of compounds and digestibility of food components could be done through processing and during mastication^19,20^. Our study observed the changes in two physical states, homogenized slurry and unhomogenized cut. The former could represent products subjected to crushing and blending, or thoroughly masticated food which involved forces strong enough to disrupt the plant cell structure and release the containing bioactive compounds. On the other hand, the latter could represent the minimally processed products which retained the tissue structure and could also be as a result of incomplete comminution during chewing. The homogenization process evidently brought damage to the cells of slurry samples which increased the surface area for immediate interactions with digestive enzymes, leading to liberation of antioxidant compounds and possibly enhancing their bioaccessibility in the gut. Unlike in unhomogenized cut samples, the intact cell wall structure could act as an effective barrier that hinders both the access of enzymes and the diffusion of the compounds out of the fragment, and therefore could lead to lower recovery of antioxidant compounds. The possibility of undetectable fracture on cell wall is high in cut sample, which means that the only way enzymes could penetrate the food is by diffusion through the cell walls. This penetration effects of digestion through layers of intact cells underlying the cut surfaces is relatively a slow process^20^. This slow release of bioactive compounds could also account to the much wider gap of increment range of cut samples than slurry state. The low recovery initially of the cut sample would mean high residual concentrations which could be released on the latter part of digestion, resulting to a higher increment range. Additionally, as reported in our previous research^53^, digesta viscosity of Saba banana showed increasing values with increase in fruit maturity and this could also affect the released rate of phenolics and antioxidant activities during simulated digestion. In the present study, higher percent increment in bio-properties of ripe stages was also observed when compared to unripe stages over the duration of simulated digestion. It seems likely that the highly viscous composition of digesta in ripe stages, particularly slurry state, could inhibit the mixing process and, as a consequence, restrict the interaction of substrates and enzymes resulting to partial release of phenolic compounds during the initial phase of digestion. The high viscosity was accounted to the different dilutions applied for each maturity stage and the presence of water-soluble pectins in ripe fruits. As already mentioned, conversion of water-insoluble to water-soluble pectin happens during ripening. This pectin from ripe fruit is capable of forming gel with water, and thus increasing the viscosity of the food matrix^54^. The effect of maturity on *in vitro* release of bioactive compounds during simulated digestion was supported by the result of correlation analysis which showed significantly negative correlation with TPC for both slurry and cut samples (*p* < 0.05).

## Conclusion

The study determined the bio-properties of Saba banana in free and bound forms which varied considerably during maturation. The different scavenging radicals used in estimating antioxidant capacities of fresh and digested samples also showed varying antioxidant potentials. The action of digestive enzymes and the changing pH conditions in the gastrointestinal tract affected the release and stability of phenolic compounds. The results of this study further demonstrate that modification in the physical structure (i.e. processing or chewing) and changes in the composition of Saba banana accompanying maturation played an important role in disintegrating plant tissue structure and regulating substrate-enzyme interaction, respectively; thus, facilitating the extraction and release of bioactive compounds from the food matrix, which in turn may have an impact on their bioaccessibility and bioavailability. For future studies, correlation of the obtained results using *in vivo* methods is necessary to better model the digestive fate of bioactive compounds in Saba banana as different physical, enzymatic, and chemical reactions and transformations are occurring during gastrointestinal digestion process.

## Methods

### Chemicals and reagents

All chemicals and reagents were of analytical grade and were obtained from various commercial sources. Sigma-Aldrich Ltd. (St. Louis, MO, USA) provided the enzymes used in simulated *in vitro* gastrointestinal digestion such as pepsin (porcine gastric mucosa, >250 U mg^−1^ solid), pancreatin (hog pancreas, 4x USP), and invertase (grade VII from baker’s yeast, >300 U mg^−1^ solid); chemicals used in antioxidant activity determination which included 2,2-diphenyl-1-picrylhydrazyl (DPPH^•^) and 2,4,6-tris(2-pyridyl)-s-triazine (TPTZ), 3-(2-pyridyl)-5,6-diphenyl-1,2,4-triazine-p,p′-disulfonic acid monosodium salt hydrate (FerroZine); FeCl_3_, and standards such as gallic acid monohydrate and (+)-catechin hydrate. Wako Pure Chemical Industries, Ltd. (Tokyo, Japan) provided FeCl_2_, FeSO_4_, 2,2′-azino-bis(3-ethylbenzothiazoline-6-sulfonic acid) (ABTS^•+^) and Trolox. Megazyme International Ltd. (Wicklow, Ireland) provided amyloglucosidase (3260 U mL^−1^) while Dojin Chemical Laboratory Co., Ltd. (Tokyo, Japan) supplied ethylenediaminetetraacetic acid disodium salt (EDTA).

### Sample and preparation

Saba bananas were purchased from Diamond Star Agro-Products Inc., Taguig City, Philippines. The fruit was received in mature green stage and was kept for ripening at 23 ± 1 °C in an incubator (MIR-153, Sanyo Electric Co., Ltd., Japan). Five maturity stages were selected based on the physicochemical properties reported in the previous study^53^. At least 7 Saba banana fingers from each of the following stages: (1) green; (2) green but turning yellow; (3) greenish yellow; (4) yellow with green tips; and (5) yellow with brown flecks, were peeled, sliced, and divided into different portions. One part was frozen in liquid N_2_, freeze-dried (Eyela FDU-1100, Tokyo Rikakikai Co. Ltd., Japan), ground, and passed through a 0.5 mm mesh sieve (Sanpo, Sanyo, Japan) for analyses of bio-properties. The remaining samples were used for simulated *in vitro* gastrointestinal digestion with varying physical states, homogenized slurry and unhomogenized cut samples. The former was homogenized using a household blender (NM200, Yamazen, China) for 2 minutes while the latter was prepared by manual and mechanical cutting (dimension of ca. <3 mm) using a food chopper (Tefal, Rumilly, France). Both states were combined with water prior to simulated digestion to have the same amount of starch content (4%).

### Extraction of free and bound phenolics

Free and bound phenolics were extracted using the method reported by Sumczynski *et al*.^55^ with slight modification. Briefly, 1 g of freeze-dried sample was treated twice with 8 mL of 80% aqueous methanol in water bath at 37 °C for 1 h. The supernatants obtained from the initial extraction and the subsequent washing were collected, combined after centrifugation at 4000 × *g* for 25 min and adjusted to pH 4.5–5.5 using 6 M HCl. The resulting solution was used to directly determine free phenolics. For bound phenolics, the residues obtained from extracting free phenolics were used. The samples were rewashed using 20 mL of distilled water. After removing water, samples were homogenized twice with 20 mL of 4 M NaOH and sonicated for 2 h in an ultrasonic bath. The pH of the mixture was then adjusted to 4.5–5.5 using 6 M HCl. Supernatant was collected after centrifugation at 4000 × *g* for 25 min.

### Determination of bio-properties

All spectrophotometric determinations were done using a Multiskan FC microplate reader (Thermo Fisher Scientific, MA, USA). Assays were performed and read in 96-well microplates. The plate contained 3 to 4 repetitions per sample and blank with 5 to 6 levels of standards.

### Total phenolic content (TPC)

TPC was determined using Folin-Ciocalteu reagent according to ISO 14502–1^56^ with some modifications. Briefly, 25 μL of extract was mixed with 125 μL of 10% (v/v) Folin-Ciocalteu reagent and 100 μL of 7.5% (w/v) Na_2_CO_3_. The mixture was allowed to stand for 1 hour in the dark at room temperature. After incubation, the absorbance was measured at 740 nm versus a prepared blank. The blank consisted of distilled water instead of sample. Gallic acid was used as a reference standard for plotting the calibration curve (0–125 ppm). The total phenolic contents were determined from the linear equation of the standard curve, expressed as mg gallic acid equivalents per 100 g of fresh weight (mg GAE 100 g^−1^ FW).

### Total flavonoid content (TFC)

TFC was determined using aluminum chloride colorimetric method^57^ with some modifications. Briefly, 33 μL of extract was diluted with 133 μL of distilled water, and 10 μL of 5% (w/v) NaNO_2_ was added. After 5 min, 10 μL of 10% (w/v) AlCl_3_ was reacted to the mixture and incubated for another 6 min. Then, 67 μL of 1 M NaOH was added and the total volume was adjusted to 333 μL by mixing 80 μL of distilled water. The mixture was mildly shaken and the absorbance was measured at 520 nm against the blank (distilled water). A calibration curve was constructed with different concentrations of catechin as standard (0–100 ppm). TFC was expressed as mg catechin equivalents per 100 g fresh weight (mg CE 100 g^−1^ FW).

### Ferric-reducing antioxidant power (FRAP)

FRAP capacity was carried out using the method of Benzie & Strain^58^ with some modifications. The sample (20 μL) was mixed with 130 μL FRAP reagent containing acetate buffer (300 mM pH 3.6), TPTZ solution (10 mM), and FeCl_3_ (20 mM) in a 10:1:1 (v/v/v) ratio. The mixture was shaken and incubated at 37 °C for 30 min away from light. The absorbance was measured at 595 nm with distilled water as blank. A calibration curve was constructed with standard solutions of FeSO_4_ (0–500 μmol/L) and the results were expressed as μmol FeSO_4_ equivalent per 100 g FW.

### Metal ion chelating (MIC) activity

MIC activity on Fe^2+^ was estimated using the method of Dinis *et al*.^59^ with some modifications. The diluted extract (300 μL) was mixed with 5 μL of 2 mM FeCl_2_ and 10 μL of 5 mM FerroZine. The mixture was gently shaken and left standing at room temperature for 10 min. Absorbance was measured at 560 nm with distilled water as blank. A standard curve of EDTA (0–30 μmol/L) was prepared and the chelating activity was expressed as μmol EDTA equivalent per 100 g FW.

### DPPH^•^ scavenging activity (DPPH)

DPPH^•^ scavenging activity was determined according to Molyneux^60^. Sample (5 μL) was mixed with 195 μL methanolic solution of DPPH^•^ (60 μM). The mixture was gently shaken, kept in the dark, and left to stand at room temperature for 30 min. Thereafter, the absorbance was measured at 520 nm against a methanol blank. The DPPH^•^ scavenging activity was calculated based on calibration curves of Trolox (0–1000 μmol/L) and Vitamin C (0–100 ppm). The results were expressed as μmol Trolox equivalent (TE) and mg Vitamin C equivalent (VCE) antioxidant capacities (TEAC and VCEAC, respectively) per 100 g FW.

### ABTS^•+^ scavenging activity (ABTS)

ABTS^•+^ scavenging activity was determined according to the method of Ketnawa *et al*.^44^ with some modifications. ABTS^•+^ solution was prepared by the reaction of 7 mM ABTS^•+^ dissolved in 2.45 mM potassium persulfate and left standing in the dark at room temperature for 12 h before use. The ABTS^•+^ solution was diluted with distilled water to obtain an absorbance of 0.7 ± 0.02 at 740 nm. The actual assay was initiated by combining 10 μL of the sample with 320 μL of diluted ABTS^•+^ solution. The ABTS^•+^ scavenging activity was measured at 740 nm after 10 min incubation at 30 °C in the dark with distilled water as blank. Same as DPPH^•^ activity, reference substances (Trolox and Vitamin C), were allowed to react with the ABTS^•+^ solution to determine TEAC and VCEAC values, respectively.

### Simulated *in vitro* gastrointestinal digestion

**Figure 5:**
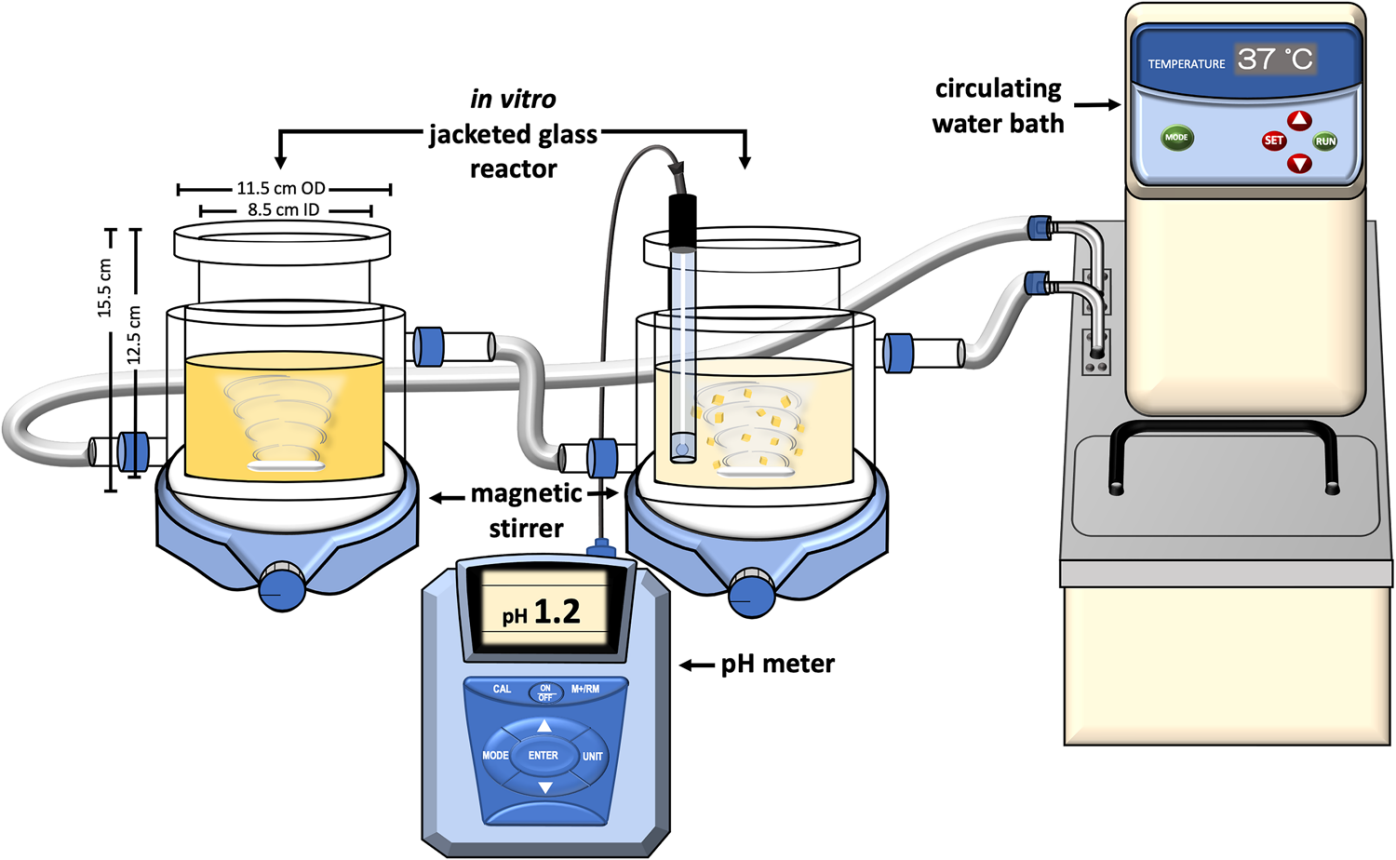
Simulated *in vitro* gastrointestinal digestion system set-up.

Two-stage simulated *in vitro* gastrointestinal digestion model as described by Ketnawa *et al*.^44^ with some modifications was employed (Fig. 5). The sample, weighing 170 g, was directly poured into a jacketed glass reactor connected to a circulating water bath (Eyela NTT-20S, Tokyo Rokakikai Co., Ltd., Japan) maintained at 37 ± 1 °C. The sample was digested for 60 min in the simulated gastric phase which was initiated by adjusting the pH to 1.2 ± 0.1 using different molar concentrations of HCl and addition of simulated gastric fluid containing 0.12 g pepsin and buffer with 0.2% (w/v) NaCl (pH adjusted to 1.2). Then, simulated small intestinal digestion phase proceeded by changing the pH to 6.8 ± 0.1 using different molar concentrations of NaOH and addition of simulated intestinal fluid containing 0.1 g pancreatin, 7.5 mg invertase, 2 mL amyloglucosidase, and buffer with 0.68% (w/v) monobasic potassium phosphate (KH_2_PO_4_) (pH adjusted to 6.8). Aliquots of mixture (1 mL) were collected at time 0 (before the start of digestion), 5 (G5), 30 (G30), and 60 min (G60) in the gastric phase; and after 5 (I5), 60 (I60), 120 (I120), 180 (I180), and 240 min (I240) in the small intestinal phase for slurry samples while cut samples was continued until 480 min (I480). Supernatants were added with 2 mL of 95% ethanol to stop the enzymatic reactions, centrifuged at 1800 × *g* for 10 min, and stored at −40 °C until further analysis. TPC and antioxidant activities were determined, the same methods as reported above.

### Statistical analysis

Statistical analysis was performed using R software version 3.3.3 GUI 1.69 Mavericks^61^. Results were presented as mean ± standard deviation. Data were analyzed using t-test and one-way analysis of variance (ANOVA) and significant differences existed between mean scores were determined using Tukey’s procedure set at *p* < 0.05. Correlation analysis based on Pearson’s method was performed among the variables.

### Ethical approval

This article does not contain any studies with human participants or animals performed by any of the authors.

## Data Availability

The research data of this study will be provided upon request.
